# Guidelines for reviewing manuscripts submitted to Animal Bioscience

**DOI:** 10.5713/ajas.20.0361

**Published:** 2020-10-14

**Authors:** Ah Reum Son, Beob Gyun Kim

**Affiliations:** 1 Department of Animal Science and Technology, Konkuk University, Seoul 05029, Korea

The objective of this editorial is to present standard guidelines in a peer review process for reviewers. This editorial contains acceptance of invitation, review process, decision for manuscripts, and confidentiality. In addition, it helps authors to publish in Animal Bioscience. This editorial does not aim to provide immutable guidelines for reviewing; however, it can assist reviewers in appropriate and effective ways.

## ACCEPTANCE OF INVITATION

When a reviewer receives an invitation to review a manuscript from the Animal Bioscience (AB) journal editorial office by e-mail with a copy of the abstract, the reviewer is expected to respond to the editorial office within five days regarding their acceptance or decline of the manuscript review. If the reviewer is not familiar with the topic of the manuscript, the reviewer has a conflict of interest with anyone of the authors or any part of the manuscript, or the reviewer is unable to complete the review within the assigned review period, the reviewer should decline the review request as soon as possible. The reviewer is welcome to recommend other appropriate reviewers.

If a reviewer accepts the invitation, the reviewer should submit the review report to the AB journal online review system within 14 days. If the reviewer requires more time to provide comments, the reviewer may request an extension of the review due date.

## REVIEW PROCESS

When reviewing a manuscript, the reviewer needs to consider whether the concept and approach are sound. While reviewing a manuscript, the following items should be considered: if the title of the manuscript accurately reflects the contents; the research objective or hypothesis is clearly presented; the manuscript is appropriately structured and clearly presented based on the Instructions for Authors; and whether the English expression is appropriate for reading and understanding the manuscript. The opinions on these items can be provided as general comments in the first part of the review report.

Most original articles published in AB are structured into sections of “Abstract,” “Introduction,” “Materials and Methods,” “Results,” and “Discussion.” A reviewer wants to check if the following requirements for each section are maintained. Critical, specific, and often negative comments will be helpful for authors to improve the quality of the manuscript. A reviewer should also remember to provide positive comments to encourage the authors.

### Abstract

The abstract should stand alone. Original articles contain a structured abstract of 300 words consisting of objectives, methods, results, and conclusions, whereas review papers contain an unstructured abstract. The abstract should provide a clear overview of the work. The result description in the abstract should be consistent with the description in the results section and be well supported by the data. The conclusion statement should represent the major findings and be supported by the results as well.

### Introduction

A clear and concise background to the experiment should be provided with sufficient citations of previous literature. The research gap should be adequately explained. The objective or hypothesis of the work should be clear and understandable.

### Materials and methods

The experimental procedures should be sufficiently described so that others can repeat the experiment. Response criteria and units used should be clearly defined. The number of replicates and animals should be sufficient for the measurements. The experimental design and statistical analysis procedures should be appropriate (see Guidelines for experimental design and statistical analysis in animal studies [1]) and the chemical analysis procedures should be appropriate. Ethical approval for animal experiments must be obtained.

### Results

The results should be described in a way that is easy and understandable. The described results should be consistent with the data presented in the tables and figures. The results of the tables and figures should be appropriate.

### Discussion and conclusions

The authors should provide sufficient and appropriate interpretations of the results. The results should be logically explained with appropriate citations. The significance of the work should be sufficiently emphasized. The conclusions should be drawn based on evidence and results.

## DECISION FOR MANUSCRIPTS

At the end of the review process, a reviewer will need to grade the manuscript for each criterion: 1) originality, 2) scientific importance, 3) experimental design, 4) adequacy of methods, and 5) brevity and clarity of the manuscript. The reviewer must choose one of four decisions: accept, minor revision, major revision, or reject. It is important to note that the reviewer should not indicate the decision of the manuscript in comments to the authors.

***Accept*** is for a manuscript requiring no change before publication. A reviewer should provide some details explaining why the manuscript should be accepted in the present form.

***Minor revision*** is for a manuscript that requires minor changes not largely involved with further biological, chemical, or statistical analyses. A reviewer wants to be clear to the authors in describing the required changes preferably with specific line numbers.

***Major revision*** is for a manuscript that requires more changes or further biological, chemical, or statistical analyses. A reviewer wants to be specific regarding the major items that require changes or improvements. If there is any inconsistency among experimental data, result descriptions, and conclusions, the decision should be major revision or rejection.

***Reject*** can be recommended when a problem in the manuscript is not fixable without running further major experiments. Any flaws in the experimental design, too many typos, grammatical errors, spelling mistakes, and journal form mismatches can also be reasons for rejection. A reviewer wants to provide constructive comments even when recommending rejection. Depending on other reviewers’ recommendations, the authors may have an opportunity to resubmit the manuscript with modifications. Detailed explanations of why the manuscript is rejected can help the authors in their future research.

## CONFIDENTIALITY

The information on the manuscript should not be disclosed before the paper is published. If any advice from colleagues is necessary, a reviewer should not reveal the contributing authors’ names and keep the manuscript details confidential.
